# Prevalence of elevated liver enzymes and its association with type 2 diabetes: A cross‐sectional study in Bangladeshi adults

**DOI:** 10.1002/edm2.116

**Published:** 2020-02-12

**Authors:** Shiful Islam, Sadaqur Rahman, Tangigul Haque, Abu Hasan Sumon, AZ Mahbub Ahmed, Nurshad Ali

**Affiliations:** ^1^ Department of Biochemistry and Molecular Biology Shahjalal University of Science and Technology Sylhet Bangladesh; ^2^ Sylhet Diabetic Hospital Sylhet Bangladesh

**Keywords:** Bangladeshi adults, liver enzymes, prevalence, type 2 diabetes

## Abstract

**Background:**

Type 2 diabetes (T2D) is a major public health concern affecting millions of people worldwide. The relationship between liver enzymes and T2D has been reported in limited studies; however, there is still a lack of evidence for the Bangladeshi population. This study aimed to evaluate the prevalence of elevated liver enzymes and examine its association with the prevalence of T2D in Bangladeshi adults.

**Methods:**

A total of 270 individuals (110 diabetic and 160 nondiabetic) were enrolled in the study. Alanine and aspartate aminotransferase (ALT, AST), alkaline phosphatase (ALP) and γ‐glutamyltransferase (GGT) activities were measured in blood serum collected from them. T2D was defined as fasting blood glucose (FBG) ≥126 mg/dL or self‐reported recent use of insulin or antidiabetic medications. Association between liver enzymes and T2D was evaluated by multinomial logistic regression analysis.

**Results:**

Overall, 61.2% of participants in T2D and 37.1% of participants in the nondiabetes group had at least one or more elevated liver enzymes. The mean concentrations of serum ALT, AST, ALP and GGT were significantly higher in the T2D group compared to the nondiabetes group. The prevalence of elevated liver enzymes was significantly higher in the diabetes group compared to the nondiabetes group (*P* < .01). In regression analysis, serum GGT activity showed an independent association with the prevalence of T2D.

**Conclusions:**

A high prevalence of elevated liver enzymes was observed in subjects having diabetes. Increased serum GGT activity was independently associated with the prevalence of T2D among Bangladeshi adults. More studies of this nature should be carried out in developing countries to get proper insights into the involvement of liver enzymes in T2D.

## Abstract 2

Elevated liver enzymes are more frequent in the diabetes group. Serum GGT is independently associated with the prevalence of type 2 diabetes.

## 1 INTRODUCTION

Diabetes mellitus is a public health threat and one of the leading causes of morbidity and mortality in the world.^1^, ^2^ According to the International Diabetes Federation (IDF), about 1 in 11 adults worldwide is affected by diabetes mellitus and over 90% of them have type 2 diabetes (T2D).^3^ About 80% of the diabetic patients live in the low‐ and middle‐income countries; in Asia, the South‐East Asian countries are particularly affected.^2^ The incidence of diabetes and associated disorders in the Bangladeshi population has increased substantially since the last decades. In Bangladesh, the number of diabetic subjects was 7.3 million in 2017, which will be increased about two times by 2045.^3^

The presence of liver disease in T2D patients has received increased attention because of their long‐term health consequences and economic burden for National Health Services.^4^ Individuals with T2D are highly prone to liver function test abnormalities than nondiabetic healthy individuals.^5^ In epidemiological studies, T2D has found to be related to different liver diseases including nonalcoholic fatty liver disease (NAFLD), liver cirrhosis and hepatocellular carcinoma.^6^, ^7^, ^8^ These liver diseases are considered as a significant contributor to death in T2D.^9^

The liver is a vital organ in metabolism that plays an important role in the regulation of glucose homeostasis.^10^, ^11^ The markers for liver dysfunction, such as alanine aminotransferase (ALT), aspartate aminotransferase (AST) and γ‐glutamyltransferase (GGT), have been shown as a good indicator to measure the liver health and involved with hepatic insulin resistance 12 and risk of T2D.^6^ ALT is considered a specific marker for liver injury and is found predominately in this organ,^11^, ^13^ while GGT is present in most cell surface and highly active in liver, pancreas and kidney.^14^ GGT mediates glutathione uptake and thought to be linked to oxidative stress and chronic inflammation,^15^, ^16^, ^17^ which are also considered the important pathways of T2D development.^11^ Thus, hepatic enzymes could be underlying biological markers connecting between liver disease and T2D.

Previously, some studies have been conducted to assess the relationship between liver enzymes and T2D in Asian,^5^, ^7^, ^11^, ^15^, ^18^, ^19^, ^20^, ^21^, ^22^, ^23^ European 4, 24, 25, 26 and American populations.^12^, ^27^, ^28^, ^29^ Most of the previous study examined the associations that included only two or three liver enzymes, and there are a limited number of studies that examined maximum number (four) of liver enzymes to evaluate the relationship with T2D. Moreover, their findings were inconsistent. The epidemiological data concerning the association between elevated liver enzymes and T2D in Bangladeshi adults are not available so far. To address these issues, we conducted a cross‐sectional study to measure the prevalence of elevated liver enzymes (ALT, AST, ALP and GGT) in nondiabetic and diabetic subjects in Bangladesh and evaluate the association of increased liver enzymes with T2D.

## 2 MATERIALS AND METHODS

### 2.1 Study area and study population

This descriptive cross‐sectional study was conducted between November 2017 and July 2018 on 110 diabetic and 160 nondiabetic participants (age range 18‐85 years) from the Sylhet region, a north‐east part of Bangladesh. The participants with T2D were enrolled from Sylhet diabetic hospital, who went there for their regular physical examinations. More than 150 patients living with diabetes were invited, among them 110 subjects participated in this study. The subjects having diabetes were confirmed by participants' self‐reported history of using of antidiabetic medications and revised criteria of the American Diabetic Association.^30^ Nondiabetic participants were selected randomly from general adults of Sylhet city region, university students, and academic and nonacademic staff members of the university. The participants with a history of hepatotoxic drug intake, alcohol intake and clinical evidence of hepatic diseases were not included in the study. All participants were informed about the study, and written consent was obtained from them before inclusion in the study. The study protocol was approved by the Internal Review Committee at the Department of Biochemistry and Molecular Biology of Shahjalal University of Science and Technology and Institutional review board of Sylhet Diabetic Hospital. All steps in Methods section were carried out following the relevant guidelines and regulations.

### 2.2 Anthropometric data

Trained medical personnel and graduate‐level students performed the anthropometric measurements according to the standard procedure described elsewhere.^31^, ^32^, ^33^, ^34^, ^35^ Individual's body weight and height were measured to calculate the body mass index (BMI), which was calculated as weight in kilogram divided by height in metres squared. Physical activity was categorized as low, medium and adequate based on participation in any activities such as jogging, bicycling, swimming or daily sports. The questionnaire also asked about the smoking habits of the participants (yes or no). Brief information on individual food habits and lifestyle was also recorded in the questionnaire form.

### 2.3 Specimen collection and laboratory measurements

The participants enrolled in the study were at least 10 hours of overnight fast before providing the blood sample. About 5 mL of the venous blood was collected in a plain dry vacutainer tube using disposable syringes. The serum sample was separated and stored at −80°C until analysis. Serum levels of glucose, triglycerides (TG), total cholesterol (TC) and albumin were measured by colorimetric methods. The enzyme activity (ALT, AST, ALP and GGT) was determined by kinetic methods. All measurements were performed using commercially available diagnostic kits (Human Diagnostic, Germany, except GGT from Vitro Scient, Egypt) with a biochemistry analyser (Humalyzer 3000, USA).

### 2.4 Definition of elevated levels of liver enzyme

Elevated liver enzymes were defined as one or more measurement of: ALT > 45 U/L in men/ >34 U/L in women, AST > 35 U/L in men/ >31 U/L in women, GGT > 55 U/L in men/ >38 U/L in women 36 and ALP > 129 U/L in men/ >104 U/L in women.^37^ Type 2 diabetes was defined according to American Diabetes Association as a fasting plasma glucose ≥ 126 mg/dL,^30^ or self‐reported recent use of insulin or antidiabetic medications.

### 2.5 Statistical analysis

Quantitative variables are expressed as mean ± SD, whereas qualitative variables are expressed by frequencies (%). Pearson's correlation coefficient test was done to assess the correlation between hepatic markers and baseline variables. Differences for the anthropometric and baseline characteristics in the gender and case‐control groups were performed by independent sample *t* test. Associations between liver enzymes and T2D were evaluated by multinomial logistic regression analysis. We used four models in the regression analysis. Model 1 was adjusted for age and sex. Model 2 was adjusted for age, sex, BMI, albumin and total protein. Model 3 was adjusted for variables used in model 1 and 2 and smoking status and physical activity. Model 4 was further adjusted for variables used in model 1 to 3 and TG and TC. A *P*‐value of <.05 was set as statistically significant. IBM SPSS software, version 23, was used for statistical data analysis.

## 3 RESULTS

### 3.1 Baseline characteristics of study subjects

**Figure 1:**
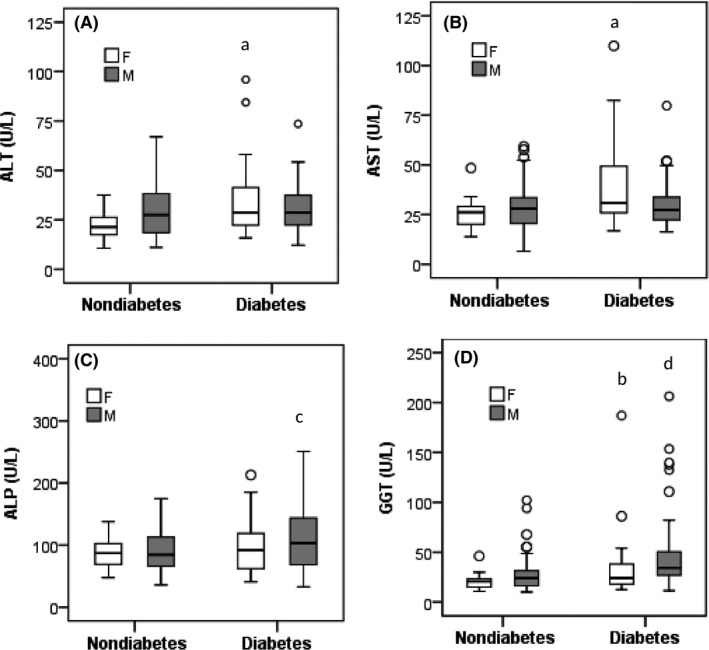
Levels of ALT (A), AST (B), ALP (C) and GGT (D) in nondiabetes and diabetes group by gender. The scale in the *y*‐axis is not similar for all liver enzymes. ^a^ *P* < .01 when comparing mean levels of ALT and AST in females with diabetes vs. females without diabetes. ^b^ *P* < .05 when comparing mean level of GGT in females with diabetes vs. females without diabetes. ^c^ *P* < .01 when comparing mean level of ALP in males with diabetes vs. males without diabetes and ^d^ *P* < .001 when comparing mean level of GGT in males with diabetes vs. males without diabetes

The baseline characteristics of the study subjects are presented in Table 1. Out of 270, 160 were nondiabetic (124 male and 36 female) and 110 were T2D participants (68 male and 42 female). The mean age for nondiabetic subjects was 36.4 ± 17.0 years and 47.2 ± 12.2 years for T2D subjects. The participants in the T2D group had a higher mean BMI (25.1 ± 3.8 kg/m^2^) than the participants in the nondiabetes group (24.0 ± 3.8 kg/m^2^). The average concentrations of ALT, AST, ALP and GGT were significantly higher in the diabetes group compared to the nondiabetes group (*P*‐value < .05 for all significant cases). Male participants in the nondiabetes group had higher concentrations of all liver enzymes than in the female participants, but a variation was observed in the T2D group (Figure 1). Male participants in the diabetes group had significantly higher mean level of ALP (*P* < .01) and GGT (*P* < .001) than males in the nondiabetes group. On the other hand, female participants in the diabetes group had higher mean levels of ALT (*P* < .01), AST (*P* < .01) and GGT (*P* < .05) than females in nondiabetes group (Figure 1). Serum levels of TC and TG were also significantly higher in the diabetes group (*P* < .001). In both groups, no significant differences were observed at the level of serum albumin, total protein, smoking status and physical activity.

**Table 1:** Baseline characteristics of the nondiabetic and diabetic participants

Variables	Nondiabetes	Diabetes	*P*‐value
N	160	110	‐
Gender [n (%)]			
Male	124 (64.6)	68 (35.4)	.000
Female	36 (46.2)	42 (53.8)	
Age (yrs)	36.4 ± 17.0	47.2 ± 12.2	.000
BMI (kg/m^2^)	24.0 ± 3.8	25.1 ± 3.8	.048
Glucose (mg/dL)	101.1 ± 13.5	212.1 ± 62.9	.000
ALT (U/L)	28.0 ± 12.7	32.4 ± 14.6	.028
AST (U/L)	27.9 ± 9.9	34.1 ± 15.7	.001
ALP (U/L)	89.3 ± 30.2	109.2 ± 50.7	.001
GGT (U/L)	25.6 ± 14.8	43.5 ± 38.2	.000
TG (mg/dL)	151.2 ± 87.2	233.1 ± 149.9	.000
TC (mg/dL)	191.0 ± 59.4	281.1 ± 119.9	.000
Albumin (mg/dL)	48.8 ± 12.1	46.1 ± 15.9	.194
Total protein (mg/dL)	78.3 ± 25.6	78.3 ± 30.5	.983
Smoking status (%)			
Yes	20.6	20.4	.979
No	79.4	79.6	
Physical activity (%)			
Low	18.7	23.5	.257
Medium	72.9	71.4	
High	8.4	5.1	

Note

Data are presented as mean ± SD. *P*‐values are obtained from independent sample *t* test in comparison between nondiabetes and diabetes group. χ^2^‐test was applied for categorical variables.

### 3.2 Prevalence of elevated liver enzymes

**Figure 2:**
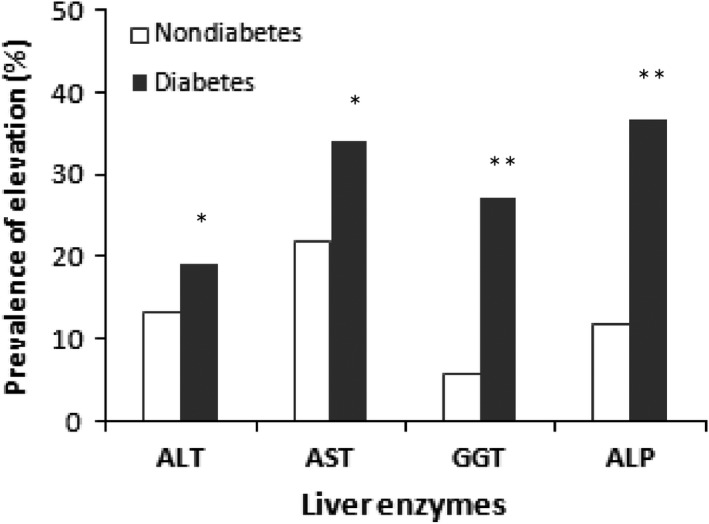
Prevalence of elevated liver enzymes (ie levels higher than the reference levels) in nondiabetes and diabetes group. *P < .01 and **P < .001 when compared to the nondiabetes group

The prevalence of elevated liver enzymes in both groups is presented in Table 2. Overall, 61.2% of participants in T2D and 37.1% of participants in the nondiabetes group had at least one or more elevated liver enzymes. The prevalence rate was significantly higher in the T2D group (ALT 19% vs 13.3%, *P* < .01; AST 34.1% vs 21.9%, *P* < .01; ALP 36.8% vs 11.9%, *P* < .001; and GGT 27.2% vs 5.7%, *P* < .001) compared to the healthy group (Table 2 and Figure 2). The prevalence of increased liver enzymes was varied in males and females in both the nondiabetes and diabetes group. In the diabetes group, the most common liver enzyme abnormalities were found among female participants than the male participants.

**Table 2:** Prevalence of elevated liver enzymes in the nondiabetes and diabetes group by gender

	Nondiabetes		Diabetes	
	Male (%)	Female (%)	Male (%)	Female (%)
ALT				
Elevated	14.1	10.0	11.1	33.3
Normal	85.9	90.0	88.9	66.7
AST				
Elevated	22.1	21.1	25.0	50.0
Normal	77.9	78.9	75.0	50.0
ALP				
Elevated	9.6	22.2	36.0	38.5
Normal	90.4	77.8	64.0	61.5
GGT				
Elevated	5.9	5.0	21.2	37.9
Normal	94.1	95.0	78.8	62.1

### 3.3 Correlation between liver enzymes and baseline variables

Table 3 presents the correlations between hepatic enzymes and baseline variables that are generally associated with diabetes. Serum AST and GGT activities showed a significant association with the age of the participants (*P* < .05). All liver enzymes showed a significant positive association with FBG concentration. Only ALP showed a significant positive association with BMI (*P* < .05). Serum ALT, AST and GGT were significantly correlated with TG and TC. The magnitude of these correlations was stronger for AST and GGT. Serum AST activity showed a significant negative correlation with albumin (*P* < .05), while ALT and GGT showed a significant positive association with total protein (*P* < .05 and *P* < .01, respectively).

**Table 3:** Correlation between hepatic markers and baseline characteristics of the participants

	ALT		AST		ALP		GGT	
	Correlation (*r*)	*P*‐value	Correlation (*r*)	*P*‐value	Correlation (*r*)	*P*‐value	Correlation (*r*)	*P*‐value
Age	.016	.822	.150	.038	.038	.613	.143	.048
BMI	.122	.090	.144	.047	.041	.578	.108	.138
Glucose	.138	.049	.196	.007	.265	.000	.224	.001
TG	.311	.000	.278	.001	.144	.079	.465	.000
TC	.180	.028	.286	.000	.134	.103	.269	.001
Albumin	−.108	.136	−.171	.018	−.030	.694	−.081	.268
Total protein	.197	.006	.023	.751	.067	.382	.168	.020

Note

Correlation was analysed using Pearson's correlation coefficient test (two‐tailed).

### 3.4 Association of liver enzymes with T2D

In multinomial logistic regression analysis, the liver enzymes showed a positive association with diabetes when covariates are adjusted in model 1 to model 3 except for AST in model 2 (Table 4). However, after adjusting lipids in model 4, only GGT activity showed a significant association with T2D (OR 1.02, 95% CI 1.00‐1.04).

**Table 4:** Association of liver enzymes with diabetes

	ALT		AST		ALP		GGT	
	OR (95% CI)	*P*‐value	OR (95% CI)	*P*‐value	OR (95% CI)	*P*‐value	OR (95% CI)	*P*‐value
Model 1	1.03 (1.00‐1.06)	.021	1.03 (1.00‐1.06)	.027	1.01 (1.00‐1.02)	.003	1.04 (1.02‐1.06)	.001
Model 2	1.03 (1.00‐1.05)	.043	1.03 (1.00‐1.06)	.060	1.01 (1.01‐1.02)	.007	1.04 (1.01‐1.06)	.001
Model 3	1.03 (1.00‐1.05)	.048	1.03 (1.00‐1.06)	.046	1.01 (1.00‐1.02)	.009	1.03 (1.00‐1.06)	.001
Model 4	1.01 (0.97‐1.06)	.548	0.99 (0.96‐1.03)	.665	1.00 (0.99‐1.02)	.181	1.02 (1.00‐1.04)	.039

Note

Multinomial logistic regression analysis was applied to evaluate the associations between liver enzymes and T2D.

Model 1: adjusted for age and sex.

Model 2: Model 1 plus BMI, albumin and total protein.

Model 3: Model 2 plus smoking status and physical activity.

Model 4: Model 3 plus TG and TC.

## 4 DISCUSSION

The association between serum liver enzymes and T2D has not been evaluated previously for the Bangladeshi population. In this case, this is the first report, which adds to the information regarding the relationship of hepatic enzymes with T2D in a Bangladeshi adult cohort. In the present study, serum activity of GGT showed an independent association with T2D.

In this study, 61.2% of participants in T2D and 37.1% of participants in the nondiabetes group had at least one or more elevated liver enzymes. The prevalence of liver enzymes (ALT, AST, ALP and GGT) above upper normal upper limit was significantly higher in subjects having diabetes. The prevalence of elevated liver enzymes in the present study (ALT 19%, AST 34.1%, ALP 36.8% and GGT 27.2%) is higher than that previously measured in a small diabetic cohort (ALT 18%, AST 3% and ALP 3%, GGT was not analysed) in Bangladesh.^38^ Moreover, the association between liver enzymes and T2D was not evaluated in that previous study. In the present investigation, the prevalence of increased liver enzymes in the T2D group was higher in female than in the male participants, which are in line with a previous study.^7^ The gender difference in elevated liver enzymes may be a reason for the individual's differences in body fat distribution and metabolism. The elevated levels of liver enzymes have been reported in Indian,^5^, ^39^ Thai,^40^ Algerian^7^ and Chinese^11^, ^19^ population. A wide variation has been observed on the prevalence of increased hepatic enzymes in these studies. Different reference values, ethnicity, age groups and demography might be considerable factors for the observed variations of these studies.

Most of the previous studies analysed ALT, AST and GGT in T2D individuals, and only a few studies included ALP. The increased levels of ALP found in our T2D individuals are consistent with previous studies where ALP was found to be elevated in diabetic subjects.^39^, ^41^ ALP in the liver was found to be associated with cell membrane, which adjoins the biliary canaliculus, and high serum levels of the liver isoenzyme indicate cholestasis rather than simply damage to the liver cells.^39^ The correlation between baseline variables and liver enzymes was evaluated in this study to see whether these variables have an influence on the concentration of the hepatic markers. In the present study, serum AST and GGT activities showed a significant correlation with age in Pearson's correlation analysis. In a previous study, increased age (>65 years) was found to be significantly associated with elevated levels of ALT, AST and GGT.^4^ Serum AST showed a positive correlation with BMI. In the current study, all liver enzymes showed a significant positive correlation with FBG concentrations. Serum ALT, AST and GGT showed a significant correlation with TG and TC, which is consistent with the findings of previous studies that have shown a strong association between liver enzymes and several factors related to metabolic syndrome.^22^, ^42^

Some studies have evaluated the relationship between liver enzymes and T2D. Most of these studies showed an independent association of GGT with T2D,^11^, ^18^, ^22^, ^24^, ^43^, ^44^, ^45^ and our results are in agreement with these findings. A follow‐up study in a Korean population also showed a strong dose‐response relationship of serum GGT concentration with the incidence of T2D.^44^ In some studies, both ALT and GGT were found to be significantly correlated with the risk of diabetes.^11^, ^18^, ^22^, ^46^ In contrast, few studies reported no significant association for ALT with diabetes when a minimum or a full range of diabetes risk factors are adjusted in the statistical models.^47^, ^48^ In the present study, ALT, ASP and ALP showed a positive association with T2D; however, the association was lost when lipid levels are adjusted in the regression models, which was consistent with the previous studies.^11^, ^21^, ^28^, ^47^, ^49^, ^50^ One possible explanation for the variability of these observations may be clarified both in terms of inadequate information of the biology of the liver enzymes and insufficient capture of their correlates and potential confounders.^22^ Moreover, ethnicity could also play some roles in this regard because of a separate analysis of the Hispanic and black subjects, no significant association was found between hepatic markers and diabetes.

The biological mechanism underlying the associations between hepatic enzymes and incidence of T2D remains unclear, some possible pathways can be considered. One is that elevated levels of ALT, AST and GGT reflect an excess fat deposition in the liver, a condition termed as NAFLD. This NAFLD is considered to be involved with metabolic syndrome, which refers to some cardiovascular risk factors related to insulin resistance, hypertension, central obesity, dyslipidaemia and T2D.^51^, ^52^, ^53^ Moreover, NAFLD is closely linked to obesity and visceral fat accumulation, and is a common feature of insulin resistance syndrome, and visceral adiposity is considered a significant contributor to T2D.^18^, ^51^ The second possibility might be serum GGT plays an important role in intracellular antioxidant defence systems, with the basic function of regulating intracellular glutathione levels.^54^ Increased oxidative stress may contribute to the development of diabetes,^55^ and chronic oxidative stress results in declined responsiveness to insulin and finally leads to T2D.^56^ Although the associated mechanism remains largely unknown, changes in inflammation that occur through oxidative stress are predicted to be a common step in the pathogenesis of T2D.^18^ In a previous study, after adjustment of C‐reactive protein, a marker of oxidative stress and inflammation, GGT showed a significant association with the risk of diabetes.^18^ The significant association between GGT and T2D observed in the present study also supports that previous study findings.^18^

One of the major strengths of the present study was the adjustment of well‐known diabetes risk factors including age, BMI, lipids, physical activities and other possible confounders to examine the relationships. Secondly, in most of the previous studies diabetes was diagnosed based on self‐reporting evidence, whereas in the current study, diabetes was diagnosed based on both self‐reporting evidence and measured FBG concentration. However, there were some limitations to the present study. Firstly, we measured the hepatic enzymes only once that may not indicate the long‐term profile. Secondly, we did not measure the hepatitis B and C infection among the participants, which could result in elevated hepatic enzymes. Thirdly, the sample size of the present study was relatively small, and we did not have information on generalizability and socio‐economic status variables. Moreover, data on antidiabetic medications and insulin resistance were not available in our study; however, the relationship remained significant in previous studies, after adjusting insulin resistance.^12^, ^28^

## 5 CONCLUSIONS

The liver enzymes showed higher activity in subjects having diabetes than subjects who do not have T2D. The most common abnormality of hepatic enzymes was found for AST, ALP and GGT. The prevalence of increased liver enzymes was higher in females than in the males in the diabetes group. Increased serum GGT activity was independently associated with T2D in Bangladeshi adults. More studies of this nature should be carried out in developing countries to get proper insights into the involvement of liver enzymes in T2D.

## CONFLICT OF INTEREST

The authors declare no conflict of interest in relation to this manuscript.

## AUTHORS' CONTRIBUTIONS

SI did the experiment, analysed the data and drafted the manuscript. SR, TH and AHS helped in sample analysis and contributed to the analysis the results. AZMH helped in sampling and contributed to revise the draft. NA played a major role in the conception and design of the study, and critical interpretation of the data, and wrote and revised the manuscript. All authors read the manuscript and approved the final version.

## ETHICAL APPROVAL

All participants were informed about the study, and written consent was obtained from them before inclusion in the study. The study protocol was approved by the Internal Review Committee at the Department of Biochemistry and Molecular Biology of Shahjalal University of Science and Technology and Institutional review board of Sylhet Diabetic Hospital. All steps in Methods section were carried out following the relevant guidelines and regulations.

## Data Availability

The data that support the findings of this study are available from the corresponding author upon reasonable request.
